# Activated PI3Kinase Delta Syndrome—A Multifaceted Disease

**DOI:** 10.3389/fped.2021.652405

**Published:** 2021-06-25

**Authors:** Romane Thouenon, Nidia Moreno-Corona, Lucie Poggi, Anne Durandy, Sven Kracker

**Affiliations:** Laboratory of Human Lymphohematopoiesis, Imagine Institute, INSERM UMR 1163, Université de Paris, Paris, France

**Keywords:** PI3K signaling, PIK3CD, PIK3R1, primary immunodeficiency, lymphoproliferation

## Abstract

Autosomal dominant gain-of-function mutations in the *PIK3CD* gene encoding the catalytic subunit p110δ of phosphoinositide 3-kinase-δ (PI3K-δ) or autosomal dominant loss-of-function mutations in the *PIK3R1* gene encoding the p85α, p55α and p50α regulatory subunits cause Activated PI3-kinase-δ syndrome (APDS; referred as type 1 APDS and type 2 APDS, respectively). Consequences of these mutations are PI3K-δ hyperactivity. Clinical presentation described for both types of APDS patients is very variable, ranging from mild or asymptomatic features to profound combined immunodeficiency. Massive lymphoproliferation, bronchiectasis, increased susceptibility to bacterial and viral infections and, at a lesser extent, auto-immune manifestations and occurrence of cancer, especially B cell lymphoma, have been described for both types of APDS patients. Here, we review clinical presentation and treatment options as well as fundamental immunological and biological features associated to PI3K-δ increased signaling.

## Introduction

**Figure 1:**
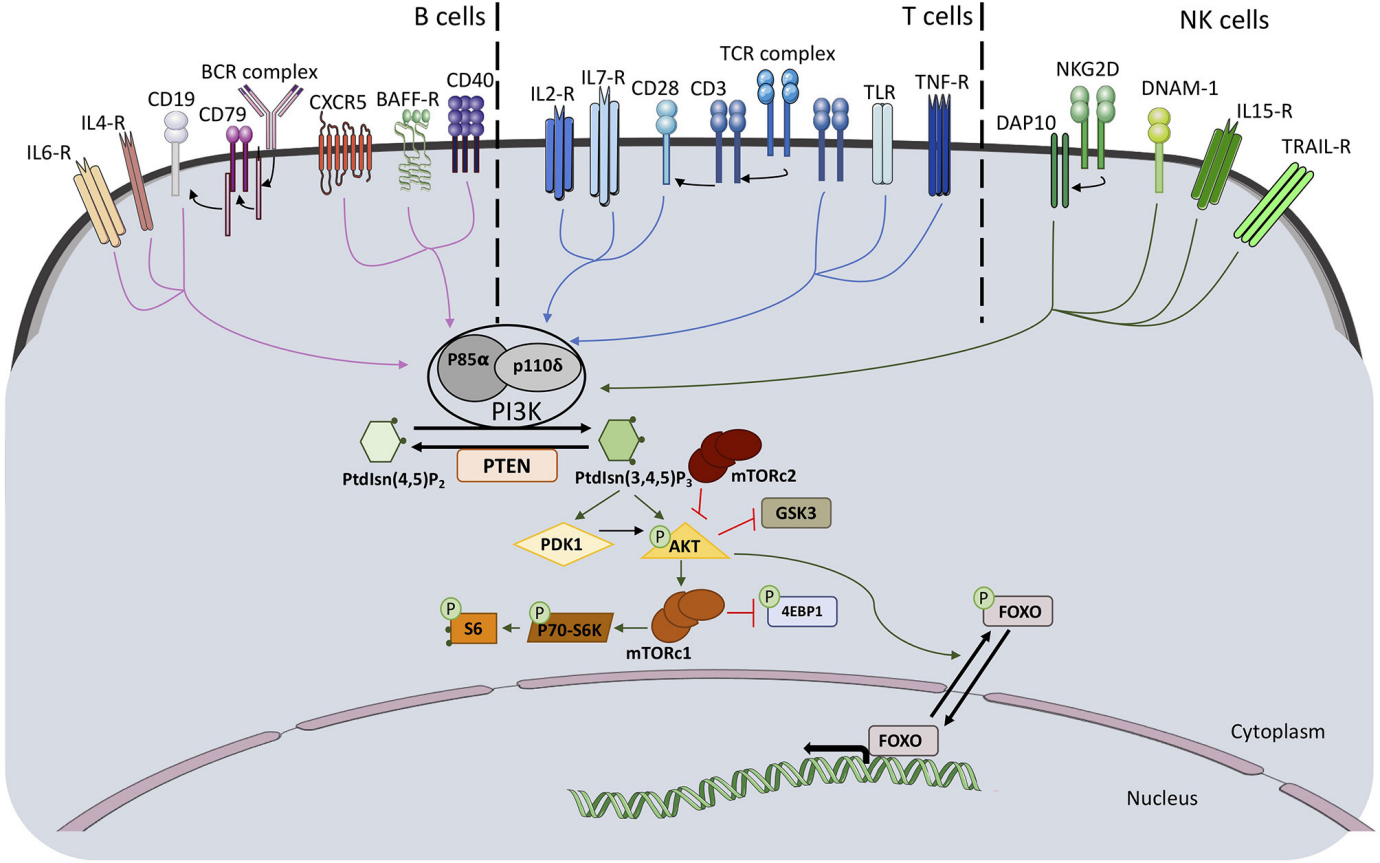
Schematic representation of PI3K pathway activation and downstream signaling molecules in B, T, and NK cells.

Class IA PI3Kinase (PI3K) molecules are composed of a p110 catalytic subunit and a regulatory subunit. The function of class IA PI3Ks is to convert phosphatidylinositol 4,5-bisphosphate into phosphatidylinositol 3,4,5-trisphosphate (PIP3), an important phospholipid secondary messenger. The genes *PIK3CA, PIK3CB* and *PIK3CD* encode for the class IA PI3K catalytic subunits p110α, p110β, and p110δ, respectively. P110δ is described to be predominantly expressed in leukocytes. Three genes encode for class1A regulatory subunits. The gene *PIK3R1* encodes due to the usage of different first exons the regulatory subunits p85α, p55α and p50α. The genes *PIK3R2* and *PIK3R3* encode each one regulatory subunit p85β and p55γ, respectively (1). Each of the catalytic subunits can bind to any of the regulatory subunits and responds to extracellular signals. The regulatory unit is required for proper activity of the catalytic unit since it regulates its stability, its cellular localization, and its kinase activity. Activation of the PI3K pathway through several membrane receptors, including T cell receptor/B cell receptor, cytokine receptors and co-stimulatory membrane molecules, lead to phosphorylation of downstream molecules, among them AKT and ribosomal protein S6 (Figure 1).

Studies of primary immunodeficient (PID) patients demonstrated the requirement of a strict balance in the PI3K pathway for optimal immune responses. On one side, bi-allelic loss-of-function (LOF) mutations in *PI3KCD* and *PIK3R1* leading to absent or decreased p110δ or p85α expression have been reported as responsible for a combined immunodeficiency or an agammaglobulinemia, respectively (2–7). On the other side, hyperactivation of the PI3Kδ pathway leads to a complex immunodeficiency: two independent reports in 2013-2014 described the identification of heterozygous missense mutations in *PIK3CD* E1021K (8)– which appears as the most frequent – and N334K, E525K (9) using whole exome sequencing (WES). They provided proof that these mutations were autosomal dominant gain-of-function (GOF) and lead to increased PI3Kδ signaling responsible for a lymphoproliferation-associated primary combined immunodeficiency syndrome (Activated PI3-kinase-δ syndrome (APDS; also referred as APDS1); OMIM: # 615513; immunodeficiency 14; IMD14; also referred as p110-delta-activating mutation causing senescent T cells, lymphadenopathy, and immunodeficiency; (PASLI also referred as PASLI-CD) (8, 9). Of note, in 2006, the heterozygous mutation E1021K in the *PIK3CD* gene had been already identified based on targeted Sanger-sequencing in DNA from a unique patient affected by an humoral immunodeficiency but without any study of the PI3K pathway (10). Following the two landmark papers, numerous case reports completed the phenotypical features and genetic changes. Further APDS1 -causing gene modifications have been described [E81K (11, 12), G124D (11, 12); R405C (13), C416R (14), Y524N (15, 16), Y524S (17), Y524D (18), E525A (19), R929C (20), E1025G (16, 21)]. Moreover, PID patients carrying additional heterozygous missense variations in *PIK3CD* possibly disease- associated have been reported (R108L) (18, 22), (R512W) (23), (P658L) (18, 24). All these missense mutations are located next to the kinase domain, in the adapter-binding domain, the linker between adapter-binding domain and RAS binding domain, the protein kinase C homology-2 domain, and the helical domain of p110 δ (25). T cell blasts isolated from APDS1 patients exhibited higher PIP3 levels before and after stimulation with antibodies to CD3 and CD28 compared with healthy controls (8). Higher levels of phosphorylated Akt and reduced levels of Foxo1 were also observed (9). Moreover, addition of p110δ inhibitor rescued this phenotype providing a further proof of increased PI3Kδ signaling.

Type 2 APDS (also referred as APDS2 and PASLI-R1); OMIM: # 616005; immunodeficiency 36; IMD36 is caused by autosomal dominant LOF mutations in *PIK3R1* (26, 27). The firstly described APDS2 mutation has been identified through WES in DNA of a PID patient presenting with a clinical and immunological phenotype reminiscent to that of APDS1 (26). Although a missense N564K variant has been reported (20), the vast majority of disease causing APDS2 mutations affect the splice donor or splice acceptor sites of exon 11 leading to an alternative splice product in which exon 11 (encoding part of the p110δ interacting domain) is deleted (28–32) (of note, the first non-coding exon was not counted in the initial description (26), thus exon skipping of coding exon 10 (in fact exon11) had been reported). The aberrant splice product enables the expression of a shortened mutant p85α (and p50α and p55α) protein lacking part of the iSH2 domain (Δ434_475) and as further consequence an hyperactivation of p110δ in APDS2 patients' lymphocytes (26, 27). Use of pharmacological PI3K delta inhibitor in lymphocytes from APDS2 patients indicated that especially the regulation of p110δ is disturbed by the mutant protein p85α^Δ434_475^. Structural studies using hydrogen-deuterium exchange mass spectrometry indicated that the APDS2 mutant protein p85α^Δ434_475^ disrupts inhibitory interactions of the nSH2, iSH2 and cSH2 domains especially within the p85α^Δ434_475^/p110δ complex, resulting in substantial basal activation of p110δ in contrast to only minimally activity of p110α within the p85α^Δ434_475^/p110α complex (33). Thus, although p85α is ubiquitously expressed, its detrimental activity is especially associated to the predominantly leucocyte expressed p110δ subunit, explaining why APDS2 resembles APDS1.

### Clinical Phenotype and Infectious Complications

Both APDS1 and APDS2 are inherited in an autosomal dominant pattern. Familial and sporadic cases associated to *de novo* mutations are documented. Paternal and maternal gonadal mosaicism has been described or suggested for APDS1 explaining puzzling inheritance pattern (35, 36). A recent report described the coexistence of uniparental disomy and the *PIK3CD* E1021K mutation resulting in cells expressing only the mutant p110δ (37).

Clinical features of both APDS1 and APDS2 are highly variable, even in the same family, and range from profound combined immunodeficiency (associated to lymphoproliferation, severe bacterial and viral infections from childhood) to isolated humoral defects Table 1. Exceptional asymptomatic patients have been reported (34). In several cohort studies, nearly all APDS patients are described as suffering from early-onset, recurrent and severe respiratory infections (38) including sinusitis, nasopharyngitis, tonsillitis, otitis media, mastoiditis, pneumonia and pulmonary empyema (14). The most common respiratory pathogens reported in both types of APDS were *Haemophilus influenzae* and *Pneumococcus pneumoniae*. However, infections with less common pathogens as Staphylococcus aureus, Pseudomonas aeruginosa, *Moraxella catarrhalis* and *Klebsiella* species were also reported (32, 34). In addition to the predominant respiratory infections, other infections affect APDS patients at lesser frequency: ocular infections, most commonly reported as (chronic) conjunctivitis but also as dacryocystitis and orbital cellulitis, have been diagnosed in several APDS patients of both types (32, 34). Skin abscesses due to Staphylococcus aureus infections have also been described (32, 34). In contrast, invasive bacterial infections are very rare (2 patients) (32). The bacterial infections reported in APDS are those commonly observed in humoral deficiencies (39).

**Table 1:** Clinical phenotype and infectious complications in APDS patients.

	**Frequency**	**References**	**Frequency**	**References**
	**APDS1**		**APDS2**	
**Infectious complications**				
**Respiratory tract infections**				
Pneumococcus pneumoniae/Haemophilus influenzae/staphylococcus aureus/Moraxella catarrhalis/pseudomonas aeruginosa	51/53	Coulter et al. (34)	23/36	Elkaim et al. (32)
**Persistent/Chronic viral infections**				
• EBV infection • HPV and VZV infection • CMV infection	14/53 11/53 11/53	Coulter et al. (34)	8/36 2/36 6/35	Elkaim et al. (32)
**Occular infections**				
• Conjonctivitis • Dacryocystitis • Orbital cellulitis	10/53 8/53 3/53 2/53	Coulter et al. (34)	7/36	Elkaim et al. (32)
Molluscum contagiosum	4/53	Coulter et al. (34)	2/36	Elkaim et al. (32)
Warts	4/53	Coulter et al. (34)	1/36	Elkaim et al. (32)
**Digestive infections**				
• Campylobacter jejuni, Salmonella typhimurium, and Clostridium difficile, • Cryptosporidium parvum	2/53	Coulter et al. (34)	1/36 1	Elkaim et al. (32) Olbrich et al. (29)
Candida	7/53	Coulter et al. (34)	3/36	Elkaim et al. (32)
Tonsillitis • With tonsillectomy	15/53 7/53	Coulter et al. (34)	13/27 10/27	Elkaim et al. (32)
**Other complications**				
Lymphadenopathy	34/53	Coulter et al. (34)	27/36	Elkaim et al. (32)
Splenomegaly	31/53	Coulter et al. (34)	15/35	Elkaim et al. (32)
Hepatomegaly	24/53	Coulter et al. (34)	8/36	Elkaim et al. (32)
Autoimmune disease	22/53	Coulter et al. (34)	6/35	Elkaim et al. (32)
Nodular mucosal lymphoid hyperplasia	17/53	Coulter et al. (34)	13/27	Elkaim et al. (32)
Enteropathy	13/53	Coulter et al. (34)	8/33	Elkaim et al. (32)
Developmental delay	12/53	Coulter et al. (34)	9/29	Elkaim et al. (32)
Malignant disease	7/53	Coulter et al. (34)	9/36	Elkaim et al. (32)
Short stature	10/53	Coulter et al. (34)	14/31	Elkaim et al. (32)

Evidence for an associated T cell and innate immune defect is provided by the frequency of viral or opportunistic infections (40): asymptomatic chronic EBV and CMV viremia (detected by PCR) has been reported for both types of APDS as well as disseminated lymphadenitis associated to CMV infections (32, 34). Persistent, severe or recurrent herpes virus infections are common in both types of APDS (41). Severe infections by Varicella zoster virus, or syncytial respiratory virus as well as molluscum contagiosum and warts, indicating pox virus and papilloma virus infections, respectively, have been reported for both types of APDS (32, 34). Chronic viral hepatitis related either to hepatitis B or C infection was reported in APDS2. Cryptosporidium parvum associated to diarrhea was reported for 2 APDS1 patients (34) whereas Giardia intestinalis was reported for 2 APDS2 patient (29, 32). Single cases of Toxoplasmosis infections were reported for both types of APDS. Episode of chronic mucocutaneous candidiasis were reported for both types of APDS patients (32, 34). Persistent granulomatous skin lesions at BCG vaccination injection sites have also been reported for both types of APDS (32, 34).

Benign lymphoproliferation manifesting as chronic or reactive lymphadenopathy, splenomegaly, hepatomegaly (typically in association) or gut infiltration is one of the whole marks for both types of APDS, reported in 75% of APDS1 and 89% of APDS2 patients (32, 34). Both types of APDS predispose to different types of B cell lymphoma (EBV+ and EBV-), especially classical Hodgkin lymphoma, diffuse large B cell lymphoma and marginal zone B cell lymphoma (8, 14, 25, 32, 42, 43).

Both types of APDS present with autoimmune manifestations, occurring in most cases after the first decade of life (44), predominantly as cytopenias and glomerulonephritis. As reported by the ESID APDS registry, 30% of APDS patients had autoimmune cytopenias, (44) such as hemolytic anemia, Evans syndrome and thrombocytopenic purpura (34). Additionally, autoimmune/inflammatory conditions reported include autoimmune thyroiditis, glomerulonephritis, sclerosing cholangitis, nephrotic syndrome, insulin-dependent diabetes, exocrine pancreatic insufficiency, autoimmune hepatitis, chronic arthritis, Sjogren syndrome, chronic eczema and autoimmune pericarditis (32, 34, 44–46).

Clinical manifestations outside of the immune system include neurodevelopmental delay presenting as mild cognitive impairment or learning disabilities reported for both types of APDS [19% (34) and 31% (32)].

A potential difference between type 1 and type 2 APDS is the notion of growth retardation more commonly associated to APDS2 (26, 32). A few reports relate APDS2 patients associated to a SHORT syndrome (47). *SHORT* syndrome is a rare genetic congenital disease characterized by *short s*tature, *h*yperextensibility, *o*cular depression, *R*ieger anomaly and *t*eething delay, with no reported immunodeficiency. Up to now, it has been described as linked to heterozygous genetic missense, nonsense and frameshift mutations in the *PIK3R1* gene located mostly downstream of exon 11 and associated to decreased PI3K activity (48–50). However, functional and structural studies for the SHORT mutation R649W located within the cSH2 of *PIK3R1* indicate that the mutation disrupts binding to phosphorylated YXXM motifs in receptor tyrosine kinases and leads as a consequence to the activation of p110α and p110δ (51). Although these observations provide functional insights for the correlation between PI3K signaling imbalance and growth retardation, the pathophysiological mechanism of APDS2 and clinical features of SHORT syndrome needs to be further elucidated.

### Disturbed B Lymphocyte Differentiation and Function in APDS

A study of a cohort of 53 APDS patients revealed variable immunoglobulin levels, with increased IgM levels (79%) and reduced total IgG levels (43%) Table 2. Fifty-eight percent of patients with normal IgG levels had, however, an IgG_2_ and IgG4 subclass deficiency (20, 34). Reduced IgA levels were common (50%), affecting mostly IgA2. Absent response to vaccination with the polysaccharide S. Pneumoniae vaccine (T -independent response) was reported in several studies (8, 20, 34). In contrast, T-dependent vaccine responses, for example, to Tetanus toxoid were found to be normal in several APDS patients (8, 20). For both types of APDS patients, peripheral blood (PB) immunophenotyping of B lymphocyte subsets indicated an increased frequency of transitional B cells (CD19+Ig(M)D+CD38+CD24+CD27– or CD20+CD10+CD27-), a reduced frequency of naïve B cells (IgM/IgD+CD27–) and of switched memory B cells (IgD–CD27+), contrasting with an increased frequency of plasmablasts [CD38++CD27++ or CD24-CD38++(IgD-CD27++sIgM-cIgM+)] compared to controls (27, 29, 53, 58). Morphological analysis of bone marrow (BM) aspirate smears from APDS1 patients revealed increased presence of immature lymphoid cells (21). Flow cytometric immunophenotyping showed a precursor B cell hyperplasia (based on CD10/CD20/CD19 expression) and impaired maturation of B lymphocytes (21). Further evaluation of the different progenitor B lymphocyte subsets in the BM of APDS1 patients suggested a block of B lymphocyte development starting at the preB-II (CD19+CD34−CD10+CD20dimIgM−) stage (52). Since increase in CD10+ B cell precursors in the BM coincided with increased CD10+ B cells in the peripheral blood of APDS1 patients (21, 52), the increased frequency of circulating immature/transitional B cells likely reflects the impaired BM development. Moreover, the normal BM development observed in an APDS1 patient after an hematopoietic stem cell transplantation (HSCT) suggests a B cell-intrinsic defect (52). The decreased numbers of naive and memory B cell subsets in contrast to increased numbers of plasmablasts in PB indicate further B cell differentiation defects outside the BM (20, 32, 34, 52). Increased frequencies of plasmablasts were also observed in lymph node biopsies from APDS2 patients (32).

**Table 2:** B and T lymphocyte dysfunction in APDS.

**Immunophenotype**					
		**APDS1 values**	**APDS1 references**	**APDS2 values**	**APDS2 references**
**Serum antibodies titers**					
IgG (IgG2 especially decreased)		Variable Normal to decreased	Coulter et al. (34) Wentink et al. (20)	Decreased	Elkaim et al. (32)
IgA		Normal to decreased	Coulter et al. (34)	Decreased	Elkaim et al. (32)
IgM		Normal to increased	Coulter et al. (34)	Normal to increased	Elkaim et al. (32)
**Vaccines responses**					
Anti-polysaccharide AB responses	Pneumococcal	Reduced to absent	Coulter et al. (34)		
Anti-peptide AB responses	Tetanus	Normal to reduced	Angulo et al. (8) Wentink et al. (20)		
**Blood B cell subsets**					
B lymphocytes	CD19+	Decreased	Coulter et al. (34) Angulo et al. (8)	Decreased	Elkaim et al. (32)
Transitional B lymphocytes	• CD19+CD27intCD38+IgM++ • CD19+IgM++CD27+ • CD10+CD27-CD20+ • CD21+CD24+CD19 +	Increased	Coulter et al. (34) Avery et al. (52) Dulau Florea et al. (21) Angulo et al. (8) Heurtier et al. (11)	Increased	Elkaim et al. (32)
Naive B lymphocytes	CD19+ CD27- IgM+ IgD-	Decreased	Avery et al. (52)	Decreased	
Marginal zone like	CD19+CD27+IgM++IgD+	Decreased	Coulter et al. (34)	Decreased	
Unswitched memory B lymphocytes	CD19+ CD27+ IgM+ IgD-	Decreased	Coulter et al. (34) Avery et al. (52) Angulo et al. (8)		
Switched memory B lymphocytes	CD19+ CD27+ IgM-IgD-	Decreased	Coulter et al. (34) Avery et al. (52) Angulo et al. (8)	Normal to decreased	Elkaim et al. (32)
Plasmablast	CD19+CD38++CD27++	Increased	Wentink et al. (20) Avery et al. (52)	Increased	Olbrich et al. (29) Martinez-Saavedra et al. (53)
**Bone marrow B cells**					
Pro B cells	CD19+CD34+CD10+CD20– IgM–	Normal	Wentink et al. (20) Avery et al. (52)		
Pre BI cells	CD19+CD34–CD10+CD20–IgM–	normal	Wentink et al. (20) Avery et al. (52)		
Pre BII cells*	CD19+CD34–CD10+CD20dimIgM–	Increased	Avery et al. (52)		
Inmature B cells	CD19+CD34–CD10+CD20+IgM+	Increased	Avery et al. (52)		
Mature B cells	CD19+CD34–CD10–CD20+	Normal	Avery et al. (52)		
**Natural killer cell subset**					
Natural Killer cells	CD3– CD16+ CD56+	Normal to decreased	Coulter et al. (34) Ruiz-Gracia et al. (54)	Normal	Elkaim et al. (32)
**Blood T cell subsets**					
Lymphocytes	CD3+	Normal	Angulo et al. (8) Coulter et al. (34)	Normal to increased	Elkaim et al. (32)
Naïve T lymphocytes	CD3+ CD4/CD8+ CD45RA+	Decreased	Angulo et al. (8) Lucas et al. (9) Bier et al. (55)	Decreased	Elkaim et al. (32)
Central Memory T lymphocytes	CD3+ CD8+ CD45RA- CCR7+	Normal	Lucas et al. (9) Edwards et al. (55)		Elkaim et al. (32) Lucas et al. (9)
Effector Memory T lymphocytes	CD3+ CD8+ CD45RA- CCR7-	Increased (expression of exhaustion and senescent markers)	Lucas et al. (9) Edwards et al. (55)	Increased	Elkaim et al. (32) Lucas et al. (9)
Effector Memory expressing CD45RA T lymphocytes	CD3+ CD8+ CD45RA+ CCR7-	Normal	Lucas et al. (9) Edwards et al. (55)	Increased	Elkaim et al. (32) Lucas et al. (9)
Central memory CD4+ T cells	CD3+ CD4+ CD45RA- CCR7+	Increased	Lucas et al. (9) Bier et al. (55)		
Effector memory CD4+T cells	CD3+ CD4+ CD45RA- CCR7-	Increased	Lucas et al. (9) Bier et al. (55)		
Circulating follicular helper T cells	CD3+ CD4+ CD45RA- CXCR5+	Increased (Th1↑)	Lucas et al. (9) Bier et al. (55)		
Circulating follicular helper T cells	CD3+ CD4+ CD45RA- CXCR5+	Increased (Th1↑)	Tsujita et al. (19) Preite et al. (56) Bier et al. (57)		
Regulatory T lymphocyte	CD41+ CD127^lo^ CD25^hi^	Normal	Lucas et al. (9) Bier et al. (57)		

Numerous patients have been firstly diagnosed as affected by an Ig class switch recombination (CSR) defect (14, 20, 32, 34, 42). In both types of APDS reduced frequencies of class-switched memory B cells were described (14, 20, 32, 34, 42). *In vitro* induced Ig CSR analyzed in different studies indicated compromised differentiation of B cells into class switched Ig (but not IgM) secreting plasmablasts. Normal and tendered to be lower expression levels of *AICDA* and normal B cell proliferation were described in *in vitro* Ig CSR cultures (9, 52). This partial Ig CSR deficiency was associated to a variable defect in the somatic hypermutation process, described to be within low-normal range on IgG and IgA transcripts (20) and normal on IgM transcripts (8).

In a mice model of Pik3cd GOF, an *in vivo* and *in vitro* defective CSR was observed associated to reduced *Aicda* mRNA expression. The addition of the PI3Kδ inhibitor leniolisib in the *in vitro* CSR cultures increased *Aicda* mRNA level and switching toward IgG1 (52). In contrast, normal affinity maturation was described in this model (52).

Analysis of sera from a cohort of APDS1 patients revealed high levels of self-reactive IgM antibodies against diverse self-antigens (59). All the analyzed patients also presented with an increased percentage of VH4-34^hi^ B cells in all subsets, suggesting increased proportions of autoreactive B cells (59). In line, the analysis of a Pik3cd GOF / SWHEL BCR transgenic murine model indicated that activated PI3Kδ-signaling impaired central and peripheral B cell tolerance mechanisms (59). Secondary C1q deficiency possibly due to the consumption of C1q driven by increased apoptotic bodies in combination with elevated IgM level observed in APDS2 patients (60) could further impair peripheral B cell tolerance.

An increased frequency of IL10-producing B lymphocytes (with a transitional B cell phenotype) was reported both in APDS1 patients' PB and in a Pik3cd GOF murine model (61) suggesting that activated PI3Kδ-signaling promotes development of B10 regulatory cells.

### T Lymphocyte Dysfunction in APDS

Immunophenotyping of APDS patients revealed CD4+ T cells lymphopenia, with a decrease in naive CD4+ and CD8+ T cells' count and a concomitant increase in effector memory CD8+ T cells' count resulting in normal to high counts of CD8^+^ T cells and a subsequent inverted CD4/CD8 ratio (8, 9, 32) Table 2.

#### CD8 T Lymphocytes

Despite the presence of EBV viremia in both types of APDS patients, EBV-specific CD8^+^ T cells were described in APDS1 patient PB. However, these cells had an effector memory phenotype (CCR7- CD45RA-) and expressed senescence-associated CD57 marker. Activation of EBV-specific CD8+ T cells showed characteristics of enhanced effector function with enhanced expression of IFNγ, Tbet and granzyme B expression compared with healthy donors' cells (9). In both non-specific and EBV-specific CD8^+^ T cells, expression of exhaustion markers (CD95, CD160, KLRG1, PD-1, 2B4) and senescence marker (CD57) was increased compared with healthy controls. Cytotoxicity of EBV specific CD8^+^ T cells against autologous EBV transformed B-LCLs was reduced (55). CD8+ effector memory T cells showed increased restimulation-induced cell death (RICD), making them more susceptible to apoptosis (55). CD57 is usually expressed by CD8+ T cells that have shortened telomeres (62). Careful study of the overall population of CD8^+^ T cells from young APDS patients of both types revealed shortened telomeres in these cells, even when CD57 was not expressed, suggesting an atypical senescent state (63). CD8^+^ T cells exhaustion and senescence phenotype have been observed in patients exhibiting chronic infection to either HIV, hepatitis B and C (64) or CMV (65) and were proposed to result from constant activation by persistent viral antigen. It is worth noting that PD-1 blockade increased virus-specific CD8+ T cell proliferation and cytokine production further indicating that exhaustion is one of the main features of APDS CD8+ T cells (66).

#### CD4 T Lymphocytes

Naive CD4+ T cells' counts were strongly reduced in both types of APDS patients as compared to healthy controls while memory CD4+ cells numbers appeared normal or increased. Treg levels were reported as unchanged (57). Among T_CM_, circulating follicular helper T cells cT_FH_ (CD45RA- CXCR5+) frequency was found to be increased (more than 3 times) in APDS patients' PB (19, 56, 57). However, the differentiation of cT_FH_ cells was reported to be skewed toward a Th1 pattern and away from a Th17 phenotype (57). The cT_FH_ -Th1 cells have been described to be inefficient at promoting B cell differentiation (67). Lymph node biopsies from both types of APDS patients indicated an important hyperplasia of T_FH_ cells (defined by expression of PD1+) present both in extrafollicular areas and germinal centers which appeared therefore disrupted by the PD1+ T cells infiltration (32, 34). Regarding the CD4+T cell compartment, analysis of cytokine production revealed an increased production of Th2 specific cytokines restricted to the memory compartment (57). Except the normal proportion of TH2 bias affecting the whole CD4+ subset, murine models are reminiscent to observations made in patients: *Pik3cd* GOF mice showed a decreased proportion of naïve CD4+ T cells and an increased proportion in CD4+ memory T cells, especially in T_FH_ cells (57). Adoptive transfer of *Pik3cd* GOF CD4+T cells in SAP-/- mice resulted in the formation of less germinal centers, suggesting that skewed differentiation toward T_FH_ results in a lower help to B cells and GC formation (57). Interestingly, BM chimeras of WT/Pik3cd GOF mice revealed more profound changes in differentiation states of CD4+ cells in the presence of *Pik3cd* GOF cells compared with control mice, suggesting that extrinsic signals drive altered differentiation of CD4+ cells (57).

### NK Cells

Although numbers of NK cells have been reported as normal or decreased in the first reports, a more careful study performed in APDS1 patients revealed both NK phenotypical and functional abnormalities, which can participate to the peculiar susceptibility of patients to viral infections (54, 68). NK phenotype was found skewed toward an immature profile, with decreased expression of CD16, CD122 and CD127 and increased expression of NKG2A Table 2. Impaired NK cytotoxicity was related to decreased conjugate formation with tumoral or antibody-coated targets, decreased ERK phosphorylation and impaired polarization of the lytic granules. Interestingly, although the NK phenotype was not modified, rapamycin treatment of patients lead to partial restoration of NK cell function and improvement of the cytolytic machinery (54).

### Therapeutic Approaches for Both Types of APDS

Treatment of both types of APDS consists mainly in prophylactic measures including long term antibiotics and Ig replacement therapy (32, 34, 69). More precise therapies have been initiated and investigated after the discovery of the genetic defects. Rapamycin (Sirolimus) treatment targeting mammalian target of rapamycin (mTOR), a downstream signaling component of the PI3K δ-signaling and a regulator of cell proliferation, was the first kind of precision therapy reported (9). Beneficial effects of rapamycin treatment were reported on both types on APDS especially by mitigating lymphoproliferation (27, 44). Less beneficial responses were noted for cytopenia and gastrointestinal symptoms (44). Two studies using orally administrated selective PI3K δ inhibitors, Leniolisib (70) and Seletalisib (71) reported reduction in lymphadenopathy and normalization of immune B cell sub-populations (reduction in the frequency of transitional B cells and a normalization of naïve B cell frequencies). Leniolisib was better tolerated in adult APDS1 patients (aged 17–31 years). Seletalisib was reported to have a favorable risk-benefit profile in a younger population (median age of APDS patients treated 15 years), even if two patients discontinued treatment due to increased hepatic enzyme considered to be drug related. Of note, however, PI3Kδ inhibitors harbor the risk to increase genomic instability in B cells by increasing AID expression and consequently mutations in off-Ig target genes, as shown with idelalisib in murine and human B cells (72).

Massive lymphoproliferation associated to life-threatening progressive combined immunodeficiency and autoimmunity are indications for HSCT (73–75). It appears as the only definitive cure for the lymphocyte mediated immune dysregulation in both types of APDS. Two case reports of HSCT patients reported similar survival rates of 9/11 and 7/9 patients, absence of long term severe graft vs. host disease and improvement of clinical manifestations (73, 74). None of the surviving HSCT patients required Ig replacement therapy by day 100 (76). However, the possible risks of transplant (adverse effects or engraftment failure) have to be compared to the benefit of available specific pharmaceutical treatments. These medical treatments could also be essential to allow disease remission and thus better opportunity for less risky HSCT procedure (75).

## Conclusion

Studies of PID patients provided valuable insights in the underlying pathophysiological mechanisms of PI3Kδ signaling and demonstrated the requirement of a strict balance in this pathway for optimal immune responses. Delineation of the molecular basis of a lymphoproliferation-associated primary combined immunodeficiency syndrome (APDS) gave evidence that hyperactive p110δ signaling impairs B cell differentiation and maturation, T cell function and homeostasis, and NK development and function. Clinical presentation and immunological abnormalities of both types of APDS are very similar although a large heterogeneity on a patient-to-patient comparison has been noticed indicating that environmental factor(s), including infections with different pathogens, as well as other genetic “modifying” factor(s) likely contribute to the disease presentation. Clinical complications such as recurrent respiratory infections, adenopathy and intestinal problems are together with frequently reported immunological abnormalities (increased IgM serum level associated to increased frequency of transitional/immature B cells and of effector/memory CD8 T cells as well as persistent CMV and/or EBV viremia) first and major diagnostic indications to consider further investigation of the PI3Kδ signaling activation. This is evaluated through the analysis of the phosphorylation status of AKT and ribosomal protein S6 or genetic examination of the APDS related genes: *PIK3CD* and *PIK3R1*.

The investigation of underlying molecular mechanisms for clinical manifestations outside of the immune system including neurodevelopmental delay described for both types of APDS and SHORT syndrome-like features particularly noted in APDS2 patients provide interesting research perspectives. Furthermore, a challenge for the future will be the identification of prognostic markers needed to guide treatment decisions. Natural history studies as the ESID-APDS registry in Europe or the Primary Immune Deficiency Treatment Consortium in North America should help to reach this goal.

## Author Contributions

RT, NM-C, LP, AD, and SK performed literature search, conceived, prepared, and wrote the mini review manuscript. All authors contributed to the article and approved the submitted version.

## Conflict of Interest

SK reports grants and payments for service agreements and travel from UCB Pharma and is a designated inventor on published patent application WO2017/198590. The remaining authors declare that the research was conducted in the absence of any commercial or financial relationships that could be construed as a potential conflict of interest.
